# Location and temporal memory of objects declines in aged marmosets (*Callithrix jacchus*)

**DOI:** 10.1038/s41598-021-88357-7

**Published:** 2021-04-28

**Authors:** Vanessa De Castro, Pascal Girard

**Affiliations:** 1 Centre de Recherche Cerveau et Cognition (CerCo), Toulouse, France; 2 Centre National de la Recherche Scientifique (CNRS) - UMR 5549, Toulouse, France; 3 Institut national de la santé et de la recherche médicale (INSERM), Toulouse, France

**Keywords:** Cognitive ageing, Learning and memory

## Abstract

Episodic memory decline is an early marker of cognitive aging in human. Although controversial in animals and called “episodic-like memory”, several models have been successfully developed, however they rarely focused on ageing. While marmoset is an emerging primate model in aging science, episodic-like memory has never been tested in this species and importantly in aged marmosets. Here, we examined if the recall of the what-when and what-where building blocks of episodic-like memory declines in ageing marmosets. We developed a naturalistic approach using spontaneous exploration of real objects by young and old marmosets in the home cage. We implemented a three-trial task with 1 week inter-trial interval. Two different sets of identical objects were presented in sample trials 1 and 2, respectively. For the test trial, two objects from each set were presented in a former position and two in a new one. We quantified the exploratory behaviour and calculated discrimination indices in a cohort of 20 marmosets. Young animals presented a preserved memory for combined what-where, and what-when components of the experiment, which declined with aging. These findings lead one to expect episodic-like memory deficits in aged marmosets.

## Introduction

Episodic memory classically refers to one’s remembering of autobiographic events, in a vivid way that involves mental time traveling^1^. Researchers strived to set up tests to apprehend episodic memory in animals because they cannot report directly autonoetic consciousness. Seminal studies of Clayton and Dickinson^2^ in jays, used the faculty of these birds to remember where they cache food and what kind of food can have decayed over time. Many following attempts used an approach evaluating how elements of episodic memory (what, where and when, or which context) need to be integrated, which lead to intense debate^3^. The difficulty of proving the sense of oneself living a past episode in animals (autonoetic experience) made it safer to use the terms «episodic-like memory»^4^. Nevertheless, some consider that it should only be called what-where-when memory^5^ because of the lack of evidence that they are reliving the episode rather than just having a sense of familiarity with it. An important family of tests in animals, as non-verbal subjects, is based on novelty preference and derived from spontaneous object recognition tests^6^. These tests aim at following one crucial feature of episodic memory, i.e. it has to be implicit without explicit training or learning. They use spontaneous recognition of objects appearing in different successive episodes^7,8^ in which rodents spontaneously explore more novel objects. Furthermore, these tests combine object, place and temporal features to determine if the animal reports whether old or recent objects have changed place across episodes^9^.

Altered episodic memory is one of the hallmarks of age-related cognitive decline, being one of first memory systems to decline in both aging pathologies like Alzheimer’s disease^10–12^ or unsuccessful non-pathological aging^13^. As pathological aging may be a slowly evolving process^14^, one strategy is to counteract it at early stages. To this end, non-human primate models are highly desirable to test prognostic factors and counteracting treatments^15,16^. Episodic memory deficits are expected to be revealed by what-where-when tests in aged animals. First, spontaneous object recognition is altered in aged rats^17^, and especially at long delays^18^ and novelty preference is affected in aged monkeys^19^. Furthermore, episodic memory tests are impacted by lesions or inactivations of prefrontal cortex and hippocampus ^138–140^, hence reflecting the deficits consecutive to alteration of these structures in human or animal cognitive aging^20–22^. Some studies have applied to human subjects similar tasks as the ones used in animals. Some were analogs to caching studies in jays^23,24^ whereas others used tasks similar to Dere’s paradigm^25–27^ in realistic environments. Both kind of tasks revealed lower performances in aged subjects^24,26^. Apart from the study by Tronche et al.^28^ in mice, to our knowledge, there are currently virtually no studies on episodic-like memory in aged animals. As non-human primates experience aging processes similar to humans^29^, we expect their episodic-like memory (ELM) to be affected by aging. While marmoset is a promising primate model of cognitive aging^30^, to date, ELM has never been tested in this species and more importantly in aged subjects. To this end, we evaluated what-where and what-when elements of ELM in a cohort of 20 marmosets including 7 old individuals. For this purpose, we used a spontaneous object recognition test (ORT), which has the advantages of being fast because no training is required, and naturalistic because measures are performed in home cages. The very few studies that have used ORT in marmosets were targeting object-location memory^31–33^ or slow and multi-trial encoding of information into long-term memory^34^. To our knowledge, we are the first to target ELM with such a test in marmosets.

## Results

### Exploratory behaviour

**Figure 1:**
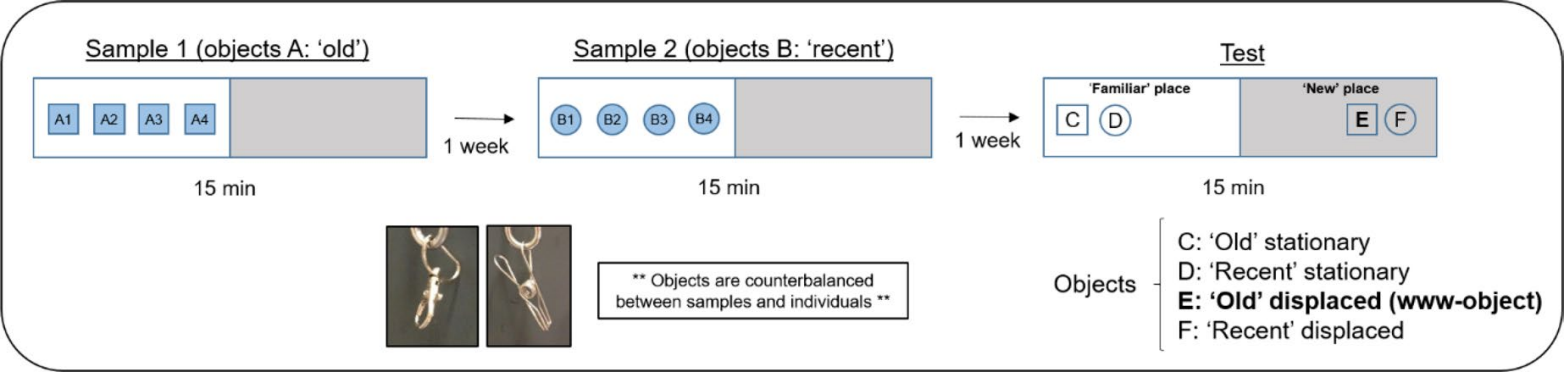
Experimental design. Animals were exposed to three different trials: sample 1, sample 2 and test; each lasting 15 min and with 1-week inter-trial interval. Two distinct sets of identical objects were presented for sample 1 and sample 2, counterbalanced across marmosets. For the test, the same objects were presented but two of them were displaced to a new position.

We used a spontaneous object exploration test to study the what-where and what-when memory components of www-memory in marmosets. This test is based on spontaneous exploratory behaviour, that is, the natural tendency to explore new objects in one’s environment^35–37^. Briefly, our version of the ORT consisted in 2 sample trials and 1 test trial with one-week inter-trial intervals, as shown in Fig. 1.

**Figure 2:**
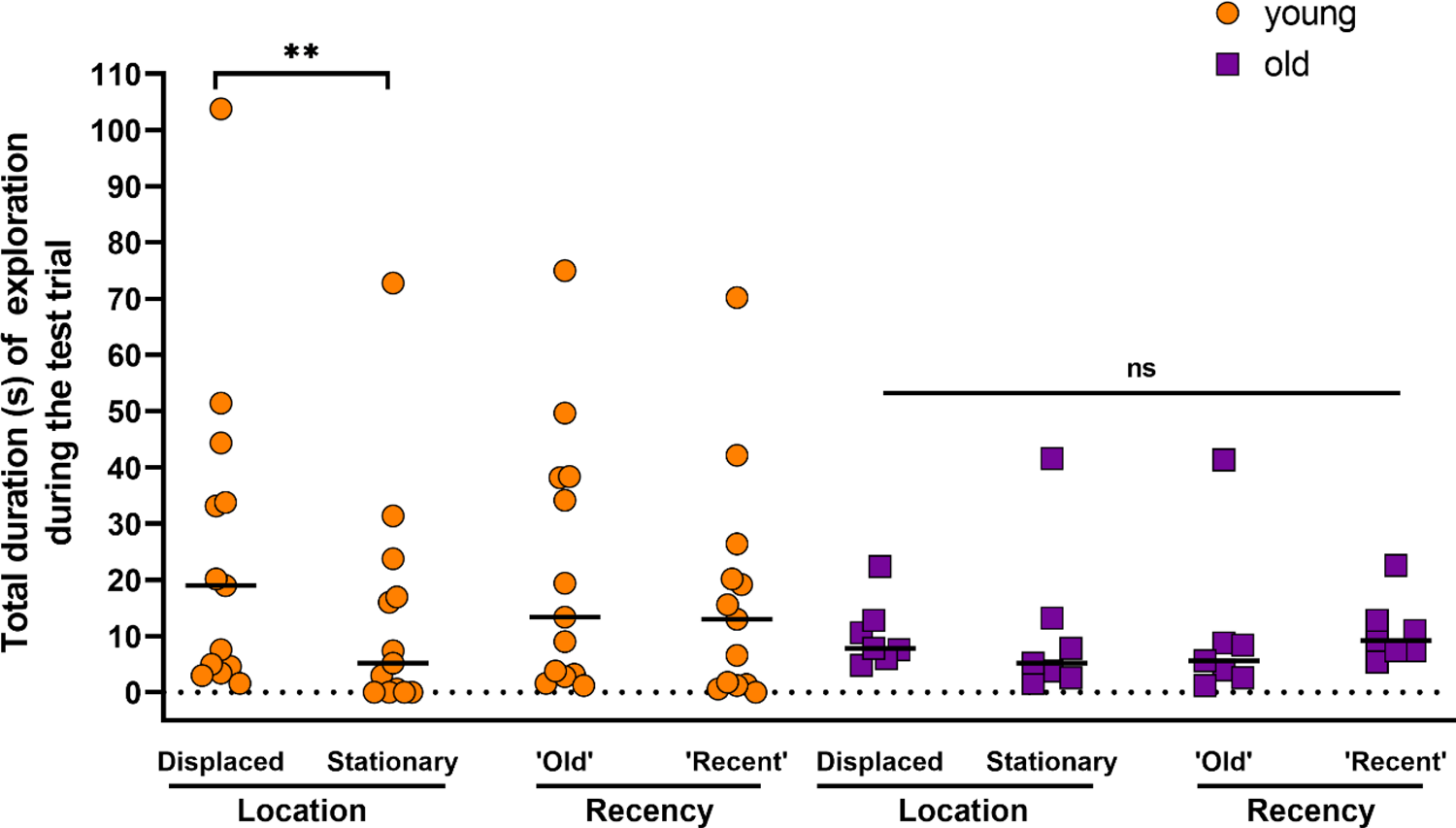
Exploratory behaviour during the test trial. Total time of exploration (duration in seconds), by the different groups (young individuals (Orange filled circle); old (Purple filled square) given by the sum of the different objects (displaced vs. stationary for location memory; and ‘old’ vs. ‘recent, for recency memory’) during the test trial. Individual values are shown. The horizontal line represents the group median (**P < 0.01, ns: not significant).

First, based on the pivotal study of Dere et al.^38^, we expect that during the test trial (1) the animals explore the objects that are displaced to a new position (‘old’ displaced and ‘recent’ displaced, E and F respectively) longer than the objects presented in the same position as in the samples trials (‘old’ stationary and ‘recent’ stationary, C and D respectively), and (2) the animals explore the objects presented in sample 1 (‘old’ objects, C and E) longer than the ones presented in sample 2 (‘recent’ objects, D and F). These exploration patterns would reflect location memory (what-where) for the former and recency memory (what-when) for the latter (see “Discrimination indices for the www-task” section). Young marmosets showed a significant difference of exploratory behaviour towards the objects (displaced and/or ‘old’) [Friedman test, $\chi$^2^(3) = 17.35, P = 0.0084]. Concretely, as shown in Fig. 2, they explore more the displaced objects (E + F) vs. the stationary objects (C + D) (P = 0.0024), while no significant difference was found between ‘old’ objects (C + E) vs. ‘recent’ objects (D + F) (P = 0.1287). Conversely, for the old group, no significant effect of exploration was found between objects (displaced and/or ‘old’) [Friedman test, $\chi$^2^(3) = 0.8824, P = 0.8297].

**Figure 3:**
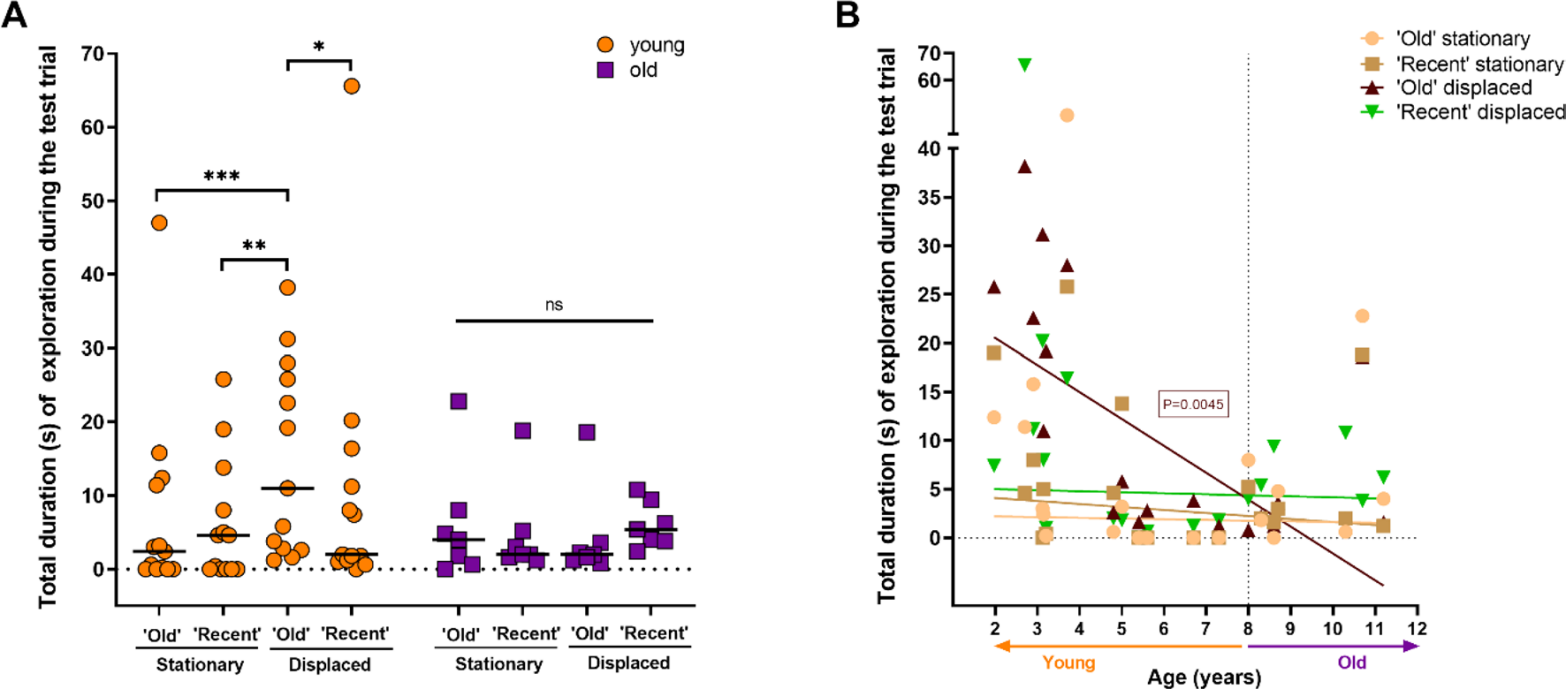
Exploratory behaviour during the test trial. (**A**) Total time of exploration (duration in seconds), by the different groups (young individuals (Orange filled circle); old (purple filled square)) of the different objects (‘old’ stationary, ‘recent’ stationary, ‘old’ displaced and ‘recent’ displaced) during the test trial. Individual values are shown. The horizontal black line represents the group median (*P < 0.05, **P < 0.01, ***P < 0.001, ns: not significant). (**B**) Individual values for total duration (in seconds) of exploration of the different objects (‘old’ stationary, ‘recent’ stationary, ‘old’ displaced and ‘recent’ displaced) during the test trial. Straight lines represent the robust linear regressions.

Secondly, we studied in more detail the exploratory pattern of our marmosets in the present design. Thus, we analyzed the total time of exploration towards the different objects (‘old’ stationary, ‘recent stationary, ‘old displaced and ‘recent’ displaced). During the test trial, young marmosets showed a significant difference of exploration between the different objects [Friedman test, $\chi$^2^(3) = 15.15, P = 0.0017]. Concretely, as shown in Fig. 3A, they explore more ‘old’ displaced vs. ‘recent’ displaced (P = 0.0227), ‘old’ displaced vs. ‘old’ stationary (P = 0.0006) and ‘old’ displaced vs. ‘recent’ stationary’ (P = 0.0018). For the other paired comparisons, no significant effects were found (‘old’ stationary vs. ‘recent’ stationary, P = 0.7613; ‘recent’ displaced vs. ‘recent’ stationary, P = 0.4034; ‘old’ stationary vs. ‘recent’ displaced, P = 0.2546). Conversely, for the old group, no significant effect of exploration was found between objects [Friedman test, $\chi$^2^(3) = 1.000, P = 0.8013]. Besides, we completed above analysis without using two age categories. Figure 3B shows a robust linear regression reveling a significant effect of age on the total duration of exploration of the ‘old’ displaced object (‘old’ stationary, P = 0.6634; ‘recent’ stationary’, P = 0.4300; ‘old’ displaced, P = 0.0045; ‘recent’ displaced, P = 0.2422). Additionally, we performed a non-linear regression to compare slopes between the objects. In agreement with the robust linear regression, the exploration of the ‘old’ displaced object significantly decreases across ages (‘old’ stationary, P = 0.0947; ‘recent’ stationary’, P = 0.9557; ‘old’ displaced, P = 0.0049; ‘recent’ displaced, P = 0.4800). More specifically, the slopes for the ‘old’ displaced and ‘recent’ displaced objects, are significantly different (P = 0.0308). No significant effect were found for the other objects’ slopes paired comparisons.

Moreover, we verified that the differences in exploratory behaviour between age groups is not due to any global unspecific impairment, such as motor and/or sensory (i.e. visual) deficits. The individual level of exploration did not differ across ages between the different trials, by applying a robust linear regression (Sample 1: P = 0.6642, Sample 2: P = 0.7932, Test: P = 0.0513, Supplementary Fig. S1). Among the few animals identified as outliers (one young in the three trials, one other young during the test and one old during the sample trials), all were spending more time than the others in exploratory behaviours. Additionally, we analyzed the duration of exploration (in seconds) of the different objects A1–A4 and B1–B4 during both samples 1 and 2, respectively. The robust linear regression (figures not shown) confirmed no significant age differences in exploration of the different objects neither for sample 1 (A1: P = 0.7578, A2: P = 0.7731, A3: P = 0.2549 and A4: P = 0.0513) nor for sample 2 (B1: P = 0.6050, B2: P = 0.7731, B3: P = 0.9935 and B4: P = 0.9935). On the other hand, no significant differences of exploration (in seconds) of the different objects were found within the age groups, neither during sample 1 [Friedman test, young: $\chi$^2^(3) = 3.554, P = 0.3138; old: $\chi$^2^(3) = 3.182, P = 0.3644], nor during sample 2 [Friedman test, young: $\chi$^2^(3) = 1.000, P = 0.8013; old: $\chi$^2^(3) = 2.652, P = 0.4484] (Supplementary Fig. S2).

**Figure 4:**
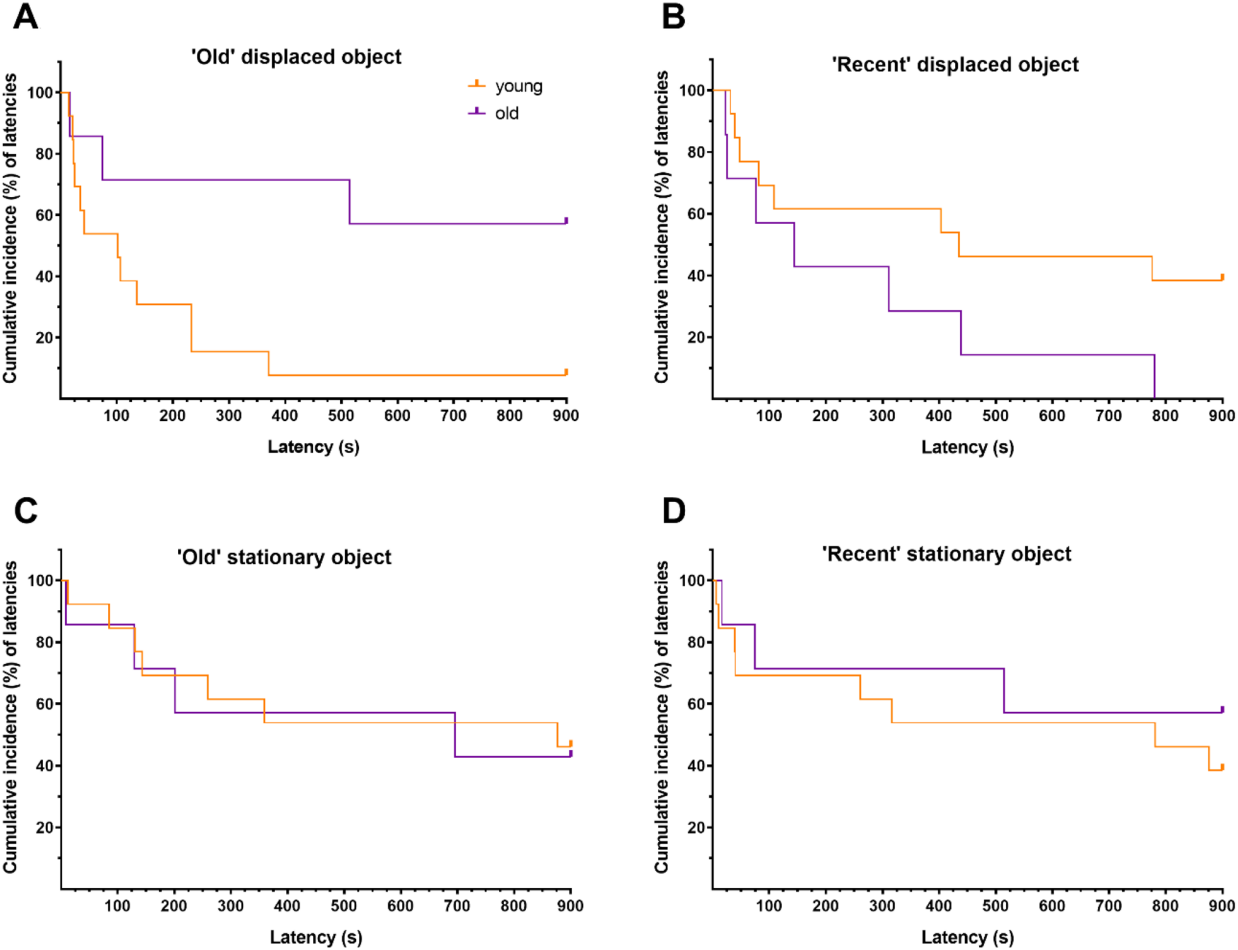
Latency. Cumulative incidence of latencies (%) over the course of the trial (900 s) to reach and explore the ‘old’ displaced object (**A**), ‘recent’ displaced object (**B**), ‘old’ stationary object (**C**) and ‘recent’ stationary object (**D**), by the different groups (young, orange; old, purple).

Finally, besides the duration of the exploratory behaviours, we also considered the latencies to reach and explore the different objects during the test trial. We were particularly interested in which object animals explore first. These latencies may in fact be a strong sign of preference because a manipulated object may progressively lose interest (novelty/preference) later within the session and the animal could start manipulating other objects^39^. Second, it is commonly accepted that aging leads to slowing down, be it general or specific to memory retrieval^40–42^. As shown in Fig. 4, we used a survival analysis that measures the percentage of animals that have explored a given object as a function of time following its presentation (i.e., latency). The latencies correspond to the time spent between the objects’ presentation and the first exploratory behaviour toward them (approach, direct gaze, manual manipulation, oral manipulation or smell manipulation; see “Methods” section for details). Younger animals were faster to reach and explore the ‘old’ displaced object. Concretely, 96.3% of the young group reached the ‘old’ displaced object faster than the old group [Hazard ratio (95% CI) 3.7 (1.330 to 10.09), Mantel-Cox test: P = 0.0247. Figure 4A]. By contrast, for the rest of the objects (‘recent’ displaced, ‘old’ stationary and ‘recent’ stationary) all animals displayed similar latencies (Fig. 4B–D). In addition, the intragroup analysis showed that for the young group, the animals reached the ‘old’ displaced object faster than the other objects (‘Old’ displaced vs. ‘recent’ displaced: P = 0.0182. ‘Old’ displaced vs. ‘recent’ stationary: P = 0.0292. ‘Old’ displaced vs. ‘old’ stationary: P = 0.0075). By contrast, the intragroup analysis for the old group showed that the animals reached faster the ‘recent’ displaced than the ‘old’ displaced (P = 0.0421) as well as the ‘recent’ displaced than the ‘recent’ stationary (P = 0.0421).

### Discrimination indices for the www-task

**Figure 5:**
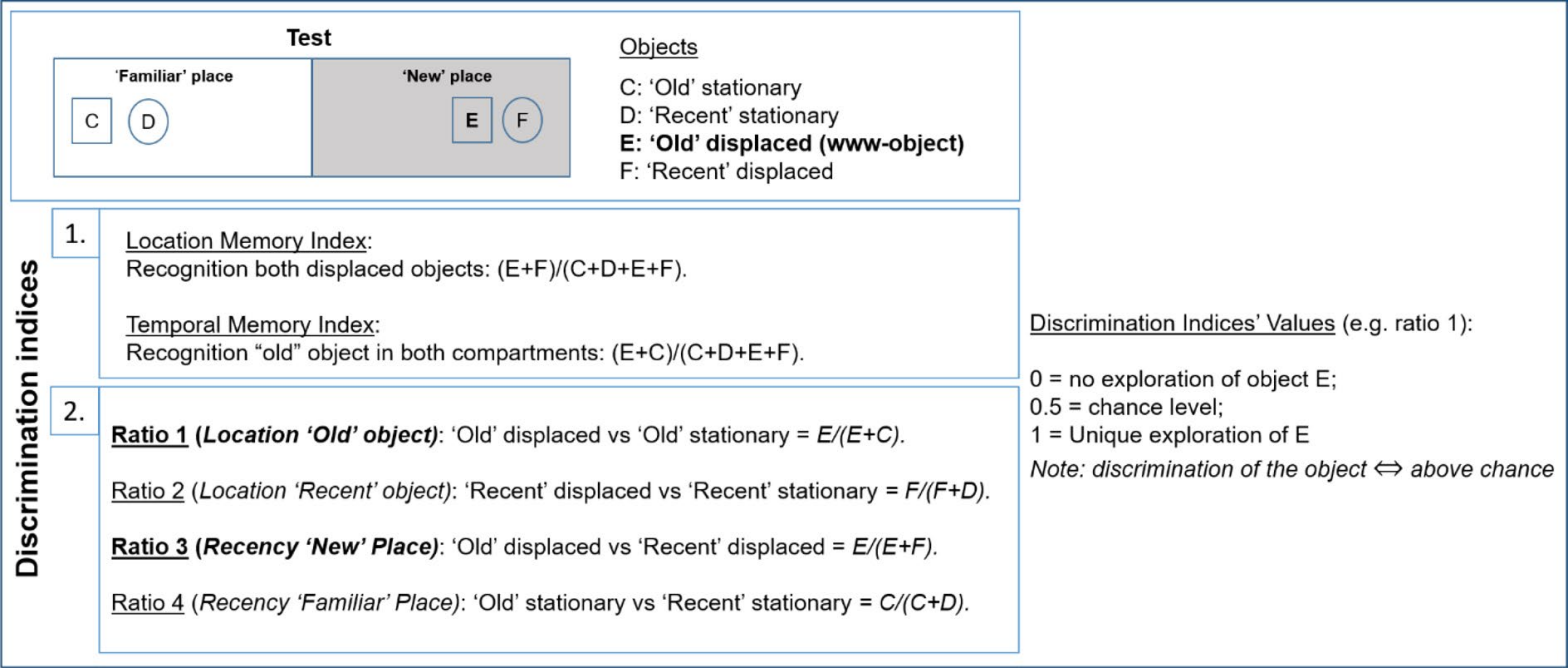
Discrimination indices. (1) Equations of the different discrimination indices of the test trial, related to temporal memory and location memory. (2) Single ratios related to the 2 × 2 location/recency design. In bold, ratios concerning the ‘old’ displaced object. ⇔: if, and only if.

Analyzing only the total exploration time could lead us to misinterpret the results in terms of memory because of disparities in inter-individual exploration levels (e.g. outliers in Fig. S1)^43^. Hence, we calculated several discrimination indices to assess what where when memory (see “Methods” section for details and Fig. 5). Besides age comparisons, the discrimination performance must be compared to chance level (no discrimination) to prove that significant discrimination between the objects does occur^43^.

As shown in Fig. 5, regarding the first prediction for exploratory behaviour, that is, a longer exploration of the displaced objects compared to the stationary objects, we computed a location memory index. The second prediction, that is, a longer exploration of the ‘old’ objects compared to the ‘recent’ objects, was tested by a temporal order memory index (recency).

**Figure 6:**
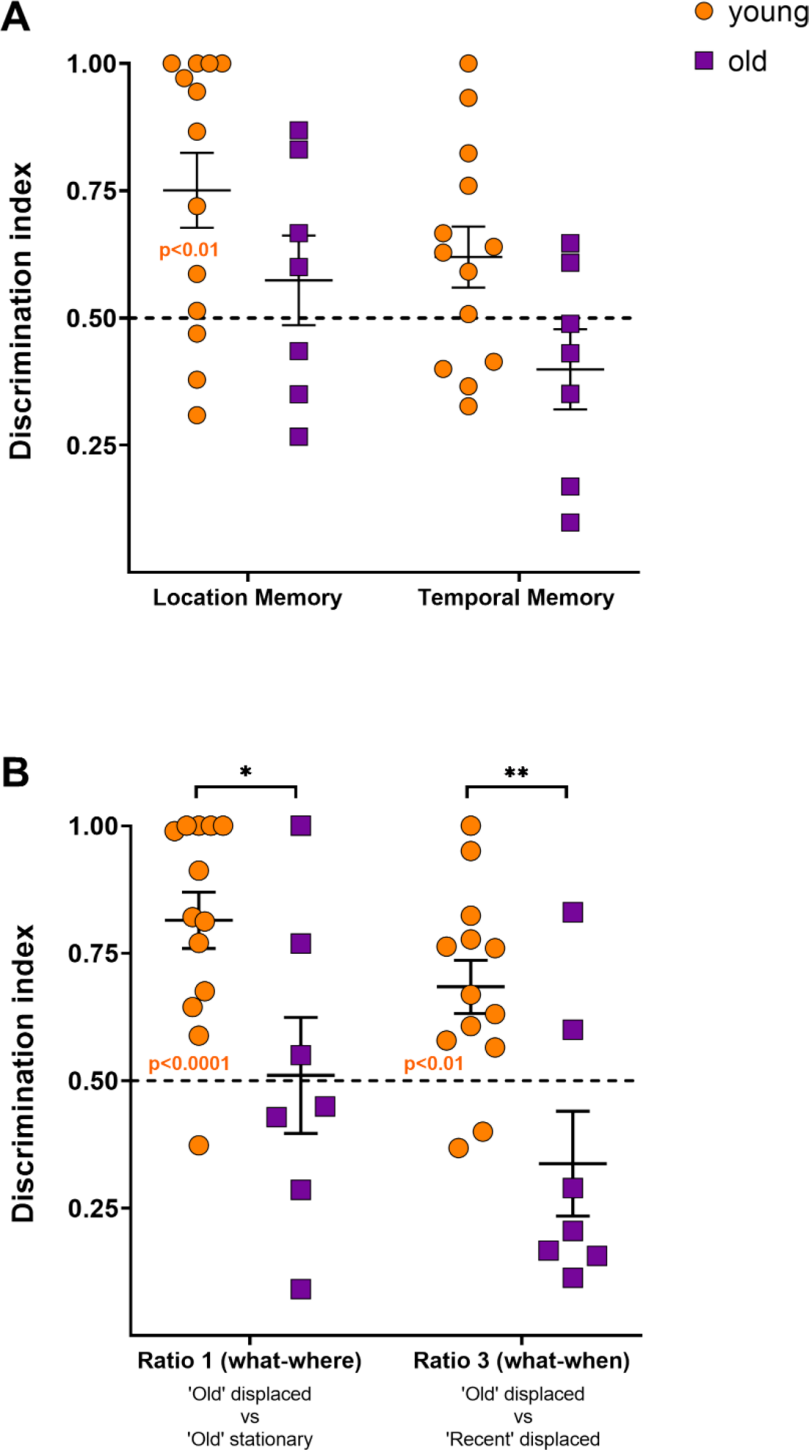
Discrimination indices related to the www-test obtained from the young and old group. (**A**) Location memory and temporal memory indices. (**B**) Discrimination indices related to the ‘old’ displaced object. Ratio 1 (‘old’ displaced vs. ‘old’ stationary), and ratio 3 (‘old’ displaced vs ‘recent’ displaced). Data are represented as means ± SEM (*P < 0.05). All individual values are shown. Dotted lines represent the chance level. Orange significant P values represent the ratio discriminations from the young group above chance level.

Location memory index in Fig. 6A revealed that young animals explored the displaced objects preferentially over the stationary ones [one sample t-test, corrected for multiple comparisons; t(12) = 3.408, P = 0.0052]. In addition, a large size effect was shown (Cohen’s d = 0.94). By contrast, the old group did not perform above chance level for this index [one sample t-test, corrected for multiple comparisons; t(6) = 0.8397, P = 0.4332]. Regarding temporal memory index, neither the young group nor the old group, preferentially explored the ‘old’ objects over the ‘recent’ ones [one sample t-test, corrected for multiple comparisons. Young: t(12) = 1.997, P = 0.0690; old: t(6) = 1.283, P = 0.2469]. On the other hand, the two-way RM ANOVA analysis showed a significant main effect of group and a tendency for types of memory [Interaction: F(1,18) = 0.08816, P = 0.7699; Types of memory: F(1,18) = 4.272, P = 0.0534, Groups: F(1,18) = 5.910, P = 0.0257]. Nonetheless, the posthoc analysis did not reveal any significant differences.

In order to further explore the results shown above and to investigate more precisely the 2 × 2 paradigm (location/recency) of the present study, we calculated single ratios, as shown in Fig. 5. Thus, we can compute location memory for both ‘old’ (ratio 1) and ‘recent’ objects (ratio 2), as well as recency memory in both ‘new’ (ratio 3) and ‘familiar’ places (ratio 4).

The young group performed above chance level for both ratio 1 (location memory for ‘old’ object) and ratio 3 (recency memory in ‘new’ place) [one sample t-test, corrected for multiple comparisons. Ratio 1: t(12) = 5.705, P < 0.0001; ratio 3: t(12) = 3.523, P = 0.0042]. In addition, a large effect size was shown for both ratios 1 and 3 (Cohen’s d, ratio 1 = 1.58; ratio 3 = 0.98). By contrast, the old group did not perform above chance level for ratio 1 or ratio 3 [one sample t-test, corrected for multiple comparisons. Ratio 1: t(6) = 0.09241, P = 0.9294; ratio 3 t(6) = 1.582, P = 0.1646]. Moreover, as shown in Fig. 6B, significant differences between young and old groups were found for both ratios 1 and 3 [two-way RM ANOVA, Groups: F(1,18) = 18.54, P = 0.0004; ratios: F(1,18) = 3.956, P = 0.0621; Interaction: F(1,18) = 0.07895, P = 0.7819. Sidak’s test, young vs. old: Ratio 1, P = 0.0151; Ratio 3, P = 0.0053].

For the rest of discrimination ratios, that is, ratio 2 (location memory for ‘recent object’) and ratio 4 (recency memory in ‘familiar’ place), neither the young group nor the old group performed above chance level [one sample t-test, corrected for multiple comparisons. Young, ratio 2: t(12) = 1.092, P = 0.2963; ratio 4: t(12) = 1.780, P = 0.1004. Old: ratio 2: t(6) = 1.143, P = 0.2965; ratio 4 t(6) = 0.3699, P = 0.7242].

## Discussion

Very few studies have assessed the building blocks ‘what, where and when’ of the ELM in non-human primates. They produced variable results depending on experimental procedures and species^44–49^. Nevertheless, these reports rely on long trainings and/or food-reward procedures. In the present study, we assessed what-where and what-when memory in aged marmosets with an object recognition test (ORT), which has the advantage of requiring no training, food reward methods and constrain. The ORT provides a valid measure of the object recognition memory and the object location memory^50^. It relies on the novelty-preference for objects (what) and places (what-where)^6,51^, and in the attractiveness for familiar objects encountered further ago in the past as a measure of temporal memory (what-when)^52^. Based on these ideas, Dere et al.^38^ developed a new paradigm combining all the above assumptions into a single exploration task, which we adapted to our research. Given the variety of designs and results’ interpretations between different authors in rodents^7,8,53–55^ together with the lack of studies in marmosets, we decided to adapt the paradigm from Trujillo-Estrada^55^, which seemed to be more appropriate for marmosets tested in their home cages. For the test trial, these behavioural protocols introduce a new position for the objects presented during the sample trials, without including novel objects. In our design, as Trujillo-Estrada et al.^55^, we introduced a completely novel position in the test scene, in contrast to the studies mentioned above where tested positions were previously present during one or both sample trials. In this regard, the difference from afore mentioned authors relies in the study of distinct types of recognition memory, for place (in our study) or for object in place (formers studies)^39^. In all cases, the location memory (what-where) capacity does not simply correspond to spatial memory (memory for ‘where’), as it requires to remember specific what-where associations (i.e. specific items in specific places)^56^. Likewise, another difference regarding the new place compared to previous works with rodents is that we choose a new panel rather than a new position above the one used in samples, because marmosets prefer to be standing up on the floor and exploring at marmoset height (see Supplementary Videos S1, S2).

First, we showed that during the experiment, marmosets interact reliably with the experimental setup. Most tested animals were able to interact with the objects during the different trials. This is not surprising given that non-deprived captive marmosets show maximum interest for novel objects in their home cage^37^. More generally, marmosets like other species of the Callithrix genus^57^ are reported to be less neophobic than other species and can even be brought to approach video setups and manipulate complex objects in the wild^58^. Furthermore and importantly, the animals did not show overt signs of motor and/or sensory (i.e. visual) impairments. However, animals from different age groups were excluded from the analysis because they failed to explore any object in one of the sample trials or during the test, or did not reach the criteria of 5 s exploration time during the samples. In this regard, in some occasions, individual marmosets explored objects only during a few seconds. In several studies using comparable episode lengths (between 5 to 10 min), marmosets explore in the same range of ours (5 to 30 s, Melamed et al.^31^, Vannucchi et al.^33^), whereas rodents explore more^7,8^. This may be the normal behavior of this primate species in such experimental setups. Video recordings showed that they were as alert as the other subjects, running and climbing in the cage. We must highlight that some of these animals were also among the lowest performers in the delayed matching-to-position task (DMTP) in Sadoun et al.^30^ (Supplementary Table S4). It is important to note that all animals, including the excluded ones, exhibited normal social, locomotor and feeding behaviours (data not shown). Then, it seems plausible to speculate that an absence of object exploration is a marker of potential deficits. It would be interesting to confirm this hypothesis by applying additional preference and motivation tests^59,60^. Moreover, a low performance in both DMTP and present task could be interpreted as a spatial attention deficit may be linked to distractibility (see discussion below), which can appear at a relatively young age^30^.

Secondly, based on Dere et al.^7^, we have seen that young animals explored the displaced objects significantly more than the stationary ones, while they did not display differential preference between the ‘old’ and the ‘recent’ ones. In contrast, old animals explored similarly these objects. In consequence, at first glance, as younger animals prefer the new location, but not ‘old’ objects, one could think they have location memory but lack recency memory. Nonetheless, the full analysis of the behavioural pattern of exploration showed that young marmosets mainly focused in the ‘old’ displaced object, while the old animals explored all objects similarly. Because our design is slightly different from others (as explained above), we think that concluding on location and recency memories does not fit precisely with our behavioural model, and cannot be generalized to our paradigm. In this sense, we think that adding together both ‘old’ objects on one side and both displaced ones on the other side, masks the details of exploration. Hence we propose a different interpretation. Saying this, we suggest that only the ‘old’ displaced object binds two main features of ELM, scilicet both spatial and temporal order of appearance, and consequently captures attention over the other objects, especially those that remained stationary^53^. Accordingly, the ‘recent’ stationary object is the less attractive object. Hence, we propose the following hypothesis to explain the exploratory pattern of young marmosets: (a) if the ‘old’ displaced object is more explored than the ‘old’ stationary one whereas there is no difference in exploration between ‘recent’ displaced and ‘recent’ stationary, it suggests that the ‘new’ place panel is not the only driving force that attracts young marmosets here but rather a what-where combination. (b) The new place is the one where there was no event before (attractive new episode, such as ‘there is something on this formerly empty wall’). If in this place, the old displaced object is more explored than the ‘recent’ displaced one, it suggests a what-when memory combination, but one cannot separate the loss of memory from a renewed interest in an old recognized object. (c) Therefore, we further hypothesized that if the ‘old’ stationary object is not more explored than the ‘recent’ stationary one in the ‘familiar’ place, it would suggest that it is not forgotten (what). All exploratory predictions are fulfilled for young marmosets, suggesting what-where and what-when memory. In this sense, we agree with Davis et al.^54^, who proposed that an increased exploration of the less recent and spatially displaced object suggests the combination of object-location and temporal information to create a what-where-when memory.

On the other hand, old marmosets did not display preferential exploration patterns. First, they could fail to discriminate between objects because they were not interested by novelty or lacked motivation. In this regard, contradictory results have been found in other monkey species^61,62^. Overall, aged marmosets displayed interest towards novelty as the total exploration time during both sample 1 and sample 2 were similar between age groups (Supplementary Table S1). Similarly, the exploration of individual objects (A1–A4 and B1–B4) during the samples were comparable across age groups (Supplementary Fig. S2). These results are in line with Insel et al.^19^, showing that aged monkeys have preference for novelty when long delay intervals are used. However, not all animals explored all the objects of the samples at the same rate. As the four objects in both samples were identical, we do not consider this mandatory as in rodent studies^53,63^. While in rodents the exploration and identification of objects are whisker-dependent^64^, in marmosets the visual system is well developed^65,66^, and in consequence we assume that exploring one object is enough to recognize all other same ones. Besides, marmosets across a large age range perform visual discrimination tasks very efficiently^30,67,68^. Nonetheless, the question remains open as to what is the minimum time of exploration necessary to remember an episode. We believe that our marmosets exploration times are compatible with www-memory (see below) and comparable to other similar marmoset studies^31^.

Strikingly, in addition to the preferential exploration, the analysis of latencies shows that young animals were faster to reach the ‘old’ displaced object, while older marmosets were significantly slower. Interestingly, old animals reach faster the ‘recent’ displaced object than the ‘old’ displaced and the ‘recent’ stationary. The clear slowing down of older marmosets is in agreement with either global, specific object processing or decision making slow-down^69–72^. In our paradigm, the animals are free to explore the objects. It is then difficult to link the slowing-down of latency to what can be observed with reaction times. Nevertheless, the difference of latencies between young and old marmosets for only one specific object argues against a global slow-down explanation. An interesting question that remains unanswered is to what extent the higher distractibility of old subjects^73–76^ could be a confounding factor. In our naturalistic setup, many congeners move and vocalize, creating a realistic situation prone to elicit distractibility and increase the cognitive charge, both known to slow down reaction times^77^. It would be tempting to explore to what extent distractibility impedes the encoding and/or recall of episodes in particular in aged marmosets and also in the lower performing young ones.

Putting all above reflections together, we propose the ‘old’ and displaced object (i.e., ‘old’ displaced) as a hallmark of what-when and what-where memory, pointing this object as possible www-memory marker (hence the term www-object). It is thus expected that the animals will explore this object preferentially if they are free of episodic-like memory deficits^78^.

Thirdly, following Dere et al.^7^ assumptions, we computed two main discrimination indices, one related to location memory and the other to temporal order memory. Here, no significant differences was found between young and old marmosets, though the young group performed above chance level for location memory. These results are consistent with the preferential exploration of displaced objects by younger animals. Hence, once again, we think that these main ratios blur conclusions about memory. Therefore, to investigate more specifically what, where and when components of ELM from our design, we calculated the objects’ relative spatial displacement and recency, splitting those main ratios into single ratios (location for ‘old’ and ‘recent’ objects, and recency for “new’ and ‘familiar’ places). According to our first hypothesis for exploratory behaviour (prediction a) we computed ratio 1 (location ‘old’ object). If animals perform above chance level for ratio 1, it means that they discriminate ‘old’ displaced object over ’old’ stationary. Consequently, ratio 1 is a location memory index for the www-object (what-where). Additionally, we calculated ratio 2 (location ‘recent’ object). If marmosets perform above chance for ratio 1 (preference for the new position of ‘old’ objects), but do not perform above chance for ratio 2 (no preference for the new position of ‘recent’ objects), it means that the recognition of the ‘old’ displaced object is not only driven by the novel position, but also by its temporal status (what-when). To confirm this, we computed ratio 3 (recency for ‘new’ place’). If animals perform above chance level for ratio 3, it means that the marmosets prefer ‘old’ over ‘recent’ objects in the ‘new place (prediction b). Finally, we calculated ratio 4 (recency in ‘familiar’ place). If marmosets do not perform above chance for ratio 4 (preference for ‘old’ in the familiar place), but do perform above chance for ratio 3 it suggests that the ‘old’ object is not forgotten (what) (prediction c). Young marmosets fulfilled above predictions for ratios, leading us to think that we obtained a measure for what-where and what-when memory.

One should note that both panels are extremely familiar as they belong permanently to the home cage, so the new place is putatively not attractive in itself but by the fact that there is a new episode in it. Then, once exploring in this new panel, they prefer the ‘old’ displaced object that binds the main www components of ELM.

The combination of these behaviours promotes the idea that samples and test may be treated as episodes by the young marmosets. In striking contrast, the aged marmosets group shows no sign of preference towards the ‘old’ displaced object. In addition, we have also observed that there is a significant decrease of exploration of ‘old’ displaced object along ages, which exploration is, at the same time, significantly different compared to the ‘recent’ stationary object. Nonetheless, we are aware that the interpretation of the what-when components is a critical issue^79^, given that in human studies it is rather seen as the “order” of events^23,25,80^, whereas in studies with animals it corresponds to ‘how long ago’ an event took place^2,81^ and then, does not necessary imply ELM^82–86^.

Some authors have suggested that what-where-when episodic-like memories are not single bits of information, but multiple components of an event bound together on trial-unique learning events^47,87^. Thus, the www-task is encoded by fragments^9^, which are combined to form what-where-when memories^88^. This combination of what-where and what-when fragments seems impaired for the old group, while it remains intact in the young group. The same holds true for humans with episodic memory deficits, who are able to remember only some details of a specific event^89^. Age impacts different components of EM (what, where and when but also how the agent interacted with the environment, how the subject recalls or imagine the past event) separately or together^90,91^. One consequence is that, in humans, aging exarcerbates and impairement recollection of episode details^92^ already present in young subjects in complex scenes^93^. It is also consistent with human naturalistic studies showing a difficulty for old subjects to bind components of episodic memory^24^. Although we observe clear group differences in discrimination performance, we cannot tentatively apply them to individuals as a ‘pathological aging diagnostic’ except for old individuals’ behaviours that would poorly obey our predictions.

At this point, could our results indicate an evidence of episodic-like memory affected by ageing in marmosets? We believe that our population of old marmosets present a deficit in combining the what, where and when memory components. Indeed, contrary to young animals, they failed to discriminate what-where and what-when (ratios 1 and 3 respectively) above chance. Moreover, there are statistical differences regarding these ratios between age groups, which are to the disadvantage of old animals. Although this suggests a deficit in ELM, we need to be cautious about this debated issue^3^. Indeed, one main unsolved point is whether marmosets clearly recollect the scenes or base their behaviour on a sense of familiarity^94^. We think our ORT protocol brings together important features to support ELM^95–97^. First, as the animals do not expect memory assessment and explore freely without expecting a reward, memory encoding could be considered as incidental^84,98,99^. Moreover, during the test, young animals use spontaneous recall, that is, not goal-directed^100^, to report scene novelty, i.e. the presence of both familiar objects in a new spatial configuration. Here, it is crucial for the marmosets to identify a particular event and distinguish it from a similar one^79^, i.e. Sample 1 from Sample 2, to perform the test according to our predictions. Second, a retention interval^87^ of 2 weeks since the presentation of the first sample, should be sufficient for a decay of the memory strength^32,33,95,101^. Conversely, considering the ability of (other species of) monkeys to remember thousands of familiar pictures^102^, one can consider that the relative familiarity of two samples may not be the primary factor marmosets rely on for the retrieval of the scenes. Finally, given our naturalistic approach, the recognition memory is also modulated by contextual information (the home-cage), which is fundamental to discriminate events using recollection, rather than familiarity^94^. Nonetheless, because we found no interaction between what-where and what-when components in our young marmosets, we cannot demonstrate an integration of www-memory, but rather consider that our results point to additive effects on building blocks necessary to come to the integration. This conclusion can be further documented by the limitations of our study, as detailed below.

We acknowledge that our study has some constraints, such as inter-individual variability, sample size, age cut-off and limited exploration time. First, we observed an important inter-subject variability that is inherent to spontaneous exploration tests^103^. Some animals seem to explore more than the others, especially in the young group but also in the old one (Figs. 2, 3). This is also well illustrated in Fig. 6 where some animals of the old age group seem to have a young pattern like in the analysis of ratios (points far above 0). The question of inter-individual variability and the noise it brings in the data interpretation could be further studied with larger cohorts (difficult to obtain) or replicating the experiments with new setups. Among the different factors of variability, aging itself is one that deserves interest^24,30,104,105^ as inter-subject variability in cognitive tasks may reflect pathological or non-successful aging for low scoring subjects^106,107^. In any case, it is widely accepted that successful aging in humans reflects a cognitive reserve supported by intact brain structures^108^. Because we used a pool of 7 aged marmosets, albeit a reasonable number for aged non-human primates, we think the study requires replication with more subjects. It would also be important to further explore the source of variability in brain structures reported to be involved in episodic memory like prefrontal cortex and medial temporal lobe structures^109–112^. Among possibilities, it is known that the medial temporal lobe tau pathology is a marker of age-related episodic-memory loss, in elderly people^113^. In this sense, there is evidence of tau hyperphosphorylation in marmosets, being more pronounced with aging^114^. Finally, because we are testing the animals in a naturalistic setting, it is also possible that the inter-individual variability comes from the difficulty of some old subjects to filter out irrelevant distractors, and to concentrate on the task as reported in humans^115^. This is a question we started to explore, the difficulty being that distractibility is not stable and would require the recording of the colony events or the addition of some controlled distracting events^116^.

Secondly, one could wonder if marmoset’s short exploration times allow for anchoring episodic memory. In our situation, as there is a direct voluntary action towards the object, we could expect this being beneficial for episodic memory because remembering performing an action is more efficient than remembering seeing a scene passively (see Hoffman et al.^117^ for a work in macaques). Furthermore, in humans, passive viewing (in comparison to a more active judgement task) is deleterious to novelty preference^118^. In human studies (where experiments are replicated), novelty preference tests show that a few seconds of interaction are enough to induce novelty preference (Fagan^119^ in infants, Pascalis and De Haan^120^ in adults, although preference may change or vanishes after some time according to experimental conditions). In a similar as Dere’s task, but using natural scenes, Kinugawa et al.^26^ used 8-s slide presentations and human subjects were able to report details (with better performances for young subjects). Ozawa et al.^121^ examining long-term memory for object location found no significant correlation (in rats) between exploration duration in sample and discrimination. However, although episode remembrance is not dependant on exploration time, the duration of the sample episode mattered (discrimination depends on long exposure, whatever the exploration time is long or not). On this topic, Albasser et al.^122^ found a positive correlation of exploration time with discrimination index, whereas Akkerman et al.^43^ and Gaskin et al.^123^ did not. Hence, this leaves open the interesting question that episodic memory may also depend on attention or distractibility.

Finally, we applied an age cutoff to distinguish young and old subjects. Although this is arbitrary, we chose the age cutoff of 8 because it is the one commonly reported around which marmosets display physical transformations and/or pathologies (diabetes, renal, bones, cardiac) that are characteristic of aging^15,124–126,141^. Considering brain aging, for instance, the age at which histological changes appear, for instance β-amyloid deposition in the brain, varies between 7 to 10 (Tardif et al.^124^) and is beyond most critical periods at which cognitive deficits start to occur (Sadoun et al.^30^).

### Conclusion and implications

Altogether, these results show for the first time, using an object recognition paradigm, that young marmosets have a preserved memory of what-where and what-when components of different real episodes, in contrast to old marmosets that seem to present a deficit in this task. Moreover, unlike older animals, the younger marmosets are able to preferentially explore the object that binds the www-elements of the task.

In conclusion, although our study approaches ELM in terms of encoding information related to its what, where and when components, we think that it is a first necessary step towards further exploration of ELM in the marmosets. We think reasonable to predict that animals that cannot “judge or feel” if something appears in this usually void place and that this “something” is the object that they did not explore for a long time, compared to another object more recently explored, would have difficulties to form episodic-like memories in terms of well separated events. With small non-human monkey cohorts in comparison with human ones, the issue of data reproducibility is unavoidable. More studies should be done in this line to confirm these results and to pursue these topics on a naturalistic approach; further developments need to include the “who” element^128^, and also turn towards object in place-context tests^129,130,79^ using, for example, computer setups in the homecage^127^.

## Methods

### Animals

Twenty marmosets (*Callithrix jacchus*) were used for the analysis, 10 females and 10 males, from ~ 2 to ~ 11 years old (Supplementary Table S3). For some analysis, animals were split into two age groups: young [(including some middle-aged animals between 5 and 7) (1.97–7.3, n = 13)] and old (8–11.2, n = 7), according to previous literature^15^. The animals were taken from our laboratory colony where they lived in social groups of 2 to 6 individuals. Each cage was enriched with nests, perches, swings, a platform and various toys. Animals were free ranging, unconstrained and without privation. Each tested animal was isolated in the upper half of the cage only during its experimental session but stayed in visual, auditory and tactile contact with its congeners. All animals were naive to the experimental procedure.

Initially 41 animals participated in this experiment: (a) 3 animals were used for the preliminary tuning of the setup; (b) twelve animals were discarded for the analysis because they did not explore any object in one of the sample trials or during the test trial, or did not reach the criteria of 5 s of exploration during the samples (Supplementary Table S4); (c) others excluded for the analysis: one because the plate that separated the cage in two parts was moved by the lower-part animals and it entered the upper part, three because there was a free marmoset in the room while the experiment was running and two because of recording problems. Hence analysis were performed on a total of 20 marmosets.

Animal housing, handling, and all experimental protocols followed the guidelines of the European Union legislation (2010/63/UE) and of the French Ministry of Agriculture (décret 2013–118). The project was approved by the National Committee for Ethical Reflection on Animal Testing following the authorization of the regional committee (authorization number: MP/03/76/11/12) and received the authorization from the French Ministry of Research (Ministère de l’Éducation Nationale, de l’Enseignement Supérieur et de la Recherche) with the authorization number : MENESR no 05215.03.

### Experimental setup

We used the spontaneous object recognition test^55^ to study the what, where and when memory in the marmosets. The experimental design consists in the presentation of 2 sample trials, followed by the test trial. One week inter-trial interval took place between each different phases. First, we exposed the animals to four identical objects and allowed them to explore freely during 15 min (Sample 1, objects ‘A’). For Sample 2, we presented a new set of four identical objects (‘B’) that were placed in the same position as in Sample 1. Finally, the test trial took place for 15 min. Here, two ‘old’ objects from Sample 1 and two ‘recent’ objects from Sample 2 were presented, so that one ‘old’ object (‘C’) and one ‘recent’ object (‘D’) were arranged in a familiar position termed stationary position (same position as in sample trials, ‘familiar’ place) while the other two objects (‘E’ and ‘F’) were moved to a novel position in the cage termed displaced position (‘new’ place) (Fig. 1). The set of ‘old’ and ‘recent’ objects were counterbalanced across marmosets. All objects were real clips and key-chains made of stainless steel to prevent material preference, to avoid destruction by the animals and to better clean odor cues. These objects had never been seen before by the 41 marmosets that participated in the experiment. Objects were chosen from a larger set that we tested on other 4 marmosets (middle-age, not used in the study) to avoid anomalously preferred or neglected objects^131^. They had roughly the same size (25 × 5 mm) and brightness (images in Fig. 1).

As soon as the subject was isolated, the experimenter placed the chains with karabiners at both ends (one to hang it from the cage and the other to hang on the object) at defined positions on a lateral wall of the cage. The stationary and displaced position were situated on the same wall, at the same height (75 mm from the floor), clearly delimited by two different panels that belong permanently to the cage (named ‘familiar’ place and ‘new’ place). Thereafter, the objects were quickly clipped to the chain’s karabiner. The animals were all familiar with the presence of chains as part of an enrichment program. The chains and objects were thoroughly cleaned with hot water after each trial. A webcam (Obqo Pro HD 960P Wi-Fi Camera A3960) was introduced inside of the cage in a Plexiglas closed box to record the behaviour of the animals during the different trials. All animals were habituated throughout 24 h to the webcam inside the box prior to the experiment. The experiment was conducted between 11.30 and 13.30. The experimenter stayed outside of the room during the 15 min exploration periods.

### Behavioural measures

We defined a behavioural catalogue for the marmosets, describing the exploratory behaviour towards the objects, divided in three categories: approach, direct gaze and manipulation (see Supplementary Videos S1, S2).

- *Approach* the subject goes towards the object and gets close to it, making direct gaze towards it, without physical contact. Cases where marmosets pass by the object while ignoring it were not considered as approaches.
- *Direct gaze* fast orientation of the head toward the object, including visual monitoring, while staying near the objects without touching them. It is assumed that free roaming marmosets are making little eye movement independently of head movements^132^. Note that we did not observe head-cocking directed to the objects^133^.
- *Manipulation* the subject actively handles the object, manually or orally (i.e. touch, bites, licks) or smells it.

The basic measure was the total time spent exploring objects during the sample phases and the test trial. The total time of exploration was defined as the sum of the different categories from the behavioural catalogue. The time spent exploring each object was analyzed observationally. The starting time for the analysis began as soon as the experimenter closed the door of the cage. The experimenter was blind to the age of the animals during this observational analysis. To exclude the possibility of an observer bias, we made an inter-observer analysis of the videos from the test trial (1/3 of the experiment), obtaining from strong (r > 0.75) to almost perfect (r ≈ 1) correlation coefficients^134^. More concretely, the inter-observer reliability for the different behaviours was: approach [Spearman r = 0.8062, n = 25], direct gaze [Spearman r = 0.7694, n = 25], manipulation (oral + smell + manual) [Spearman r = 0.9936, n = 25] and total time of exploration (approach + direct gaze + manipulation) [Spearman r = 0.9742, n = 25]. In addition, we calculated the inter-observer correlation for each subject considering all the different behaviours towards the distinct objects (Supplementary Table S5). Videos analysis and behavior coding was performed with the free software Solomon Coder (beta version 17.03.22). This software allows analyzing the videos frame by frame, giving the option to slow down the recordings to observe in detail the behaviour of the animal.

Additionally, the total time of exploration of the different objects was used to calculate different discrimination indices (depicted in Fig. 5). (1) Two main indices based on Dere et al.^135^: location memory index (recognition both displaced objects): (E + F)/(C + D + E + F) and temporal memory index (recognition ‘old’ object in both compartments): (E + C)/(C + D + E + F). (2) Additional indices were computed according to our protocol that uses a 2 × 2 recency/location. Concretely, ratio 1 (location ‘old’ object) is an index of discrimination of the ‘old’ displaced over the ‘old’ stationary objects [E/(E + C)], ratio 2 (location ‘recent’ object)] is an index of discrimination of the ‘recent’ displaced over the ‘recent’ stationary objects [F/(F + D)], ratio 3 (recency ‘new’ place) is an index of discrimination of the ‘old’ displaced over the ‘recent’ displaced objects in the ‘new’ place’ [E/(E + F)], and ratio 4 (recency ‘familiar’ place) is an index of discrimination of the ‘old’ stationary over the ‘recent’ stationary objects in the ‘familiar’ place [C/(C + D)]. The combination of these 4 ratios will give us a measure of www-memory (see “Discussion” section).

### Statistical analysis

Firstly, to evaluate the effect of age on exploratory behavior, we analyzed the time spent exploring the objects (total duration in seconds) during the different three trials (sample 1, sample 2 and test), as well as the exploration of the different objects within a trial, applying a robust linear regression, because of the presence of outliers. The presence of outliers was detected by the ROUT method^136^, as implemented in the statistic software GraphPad Prism 8.0.0. Group analysis for exploratory behaviour (sample and test trials) was performed using the non-parametrical Friedman test, because the data did not cover the assumptions of normality and heteroscedasticity. When a significant effect was observed, we used a False Discovery Rate correction (two-stage step-up method of Benjamini, Krieger and Yekutieli) according to the number of conditions for multiple comparisons. Finally, to complete the analysis from the test trial while avoiding splitting data by age groups, we applied a non-linear regression in order to compare slopes.

To study the what, where and when components of the episodic-like memory, we analyzed the discrimination indices obtained from the test phase. First, to verify values of the discrimination indices were above chance level (0.5), we applied one-sample t-tests corrected for multiple comparisons [Bonferroni correction ($\frac{p \;value}{{number\; of\; tests }} =$ $\frac{0.05}{8}$ ⇒ *alpha corrected* = 0.0125 for location and temporal memory indices and alpha corrected = 0.00625 for single ratios]. Second, if the results were significant, we calculated the effect size applying Cohen’s d formula for one-sample t-test [Cohen’s d = $\frac{m - \mu }{s}$, where ‘*m’* is the sample mean, ‘µ’ is the theoretical mean against which the mean of our sample is compared (0.5) and ‘*s*’ is the sample standard deviation with n − 1 degrees of freedom]. Finally, to compare ratios of interest between age groups, we used a Two-Way Repeated Measures ANOVA (ratios × groups) because the discrimination of the components “when” and “where” cannot be dissociated from the “what” component^53^, followed by a post-hoc Sidak test.

For the study of the latencies, we used a survival analysis based on cox regression modelling and Kaplan Meier estimates^137^. This method prevents to arbitrarily assign the maximum trial duration to the latency when an animal did not explore an object, as this is a source of bias and misleading conclusions. Hence, we treated an omitted behaviour like a censored observation (i.e. similar to failures in survival data). Finally, we applied the Spearman Rank Correlation Coefficient for the inter-observer analysis.

All individual values are shown in the graphs. The data for the linear analysis of the exploratory behaviour are represented as linear regression fit scatter plots. The exploratory behaviour analysis are represented by scatter plots (group median). The discrimination indices are represented by scatter plots (mean ± SEM). The latencies are represented by cumulative incidence plots. All analysis and graphs were performed using GraphPad Prism version 9.1.0 for Windows 64-bit, GraphPad Software, San Diego, California USA. The significance level was set at 95% of confidence.

## Supplementary Information

Supplementary Information 1.

Supplementary Video S1.

Supplementary Video S2.

## Data Availability

The datasets generated during and/or analyzed during the current study are available from the corresponding authors on reasonable request.
