# Supplementary material for: Location and temporal memory of objects declines in aged marmosets (Callithrix jacchus)
Source: Sci Rep. 2021 Apr 28;11:9138. doi: 10.1038/s41598-021-88357-7 (PMC8080792; doi:10.1038/s41598-021-88357-7)
Supplement: Supplementary file 1 — Supplementary Information 1. [file 41598_2021_88357_MOESM1_ESM.pdf]

Supplementary Information:

Location and temporal memory of objects declines in aged marmosets (*Callithrix jacchus*).

Authors:

Vanessa De Castro [1,2*], Pascal Girard [1,3,*].

Affiliations:

[1] Centre de Recherche Cerveau et Cognition (CerCo), Toulouse (France).

[2] Centre National de la Recherche Scientifique (CNRS) - UMR 5549, Toulouse (France).

[3] Institut national de la santé et de la recherche médicale (INSERM), Toulouse (France).

*Correspondence:

Vanessa De Castro: vanessa.decastro@cnrs.fr

Pascal Girard: pascal.girard@cnrs.fr

Supplementary Figure S1. Exploratory behaviour in the www-task.

Supplementary Figure S2. Exploratory behaviour during the sample trials.

Supplementary Table S1. Duration of exploration during the sample trials.

Supplementary Table S2. Duration of exploration during the test trial.

Supplementary Table S3. Subjects kept for the statistical analysis.

Supplementary Table S4. Subjects excluded from the statistical analysis.

Supplementary Table S5. Subjects' inter-observer correlations during the test trial.

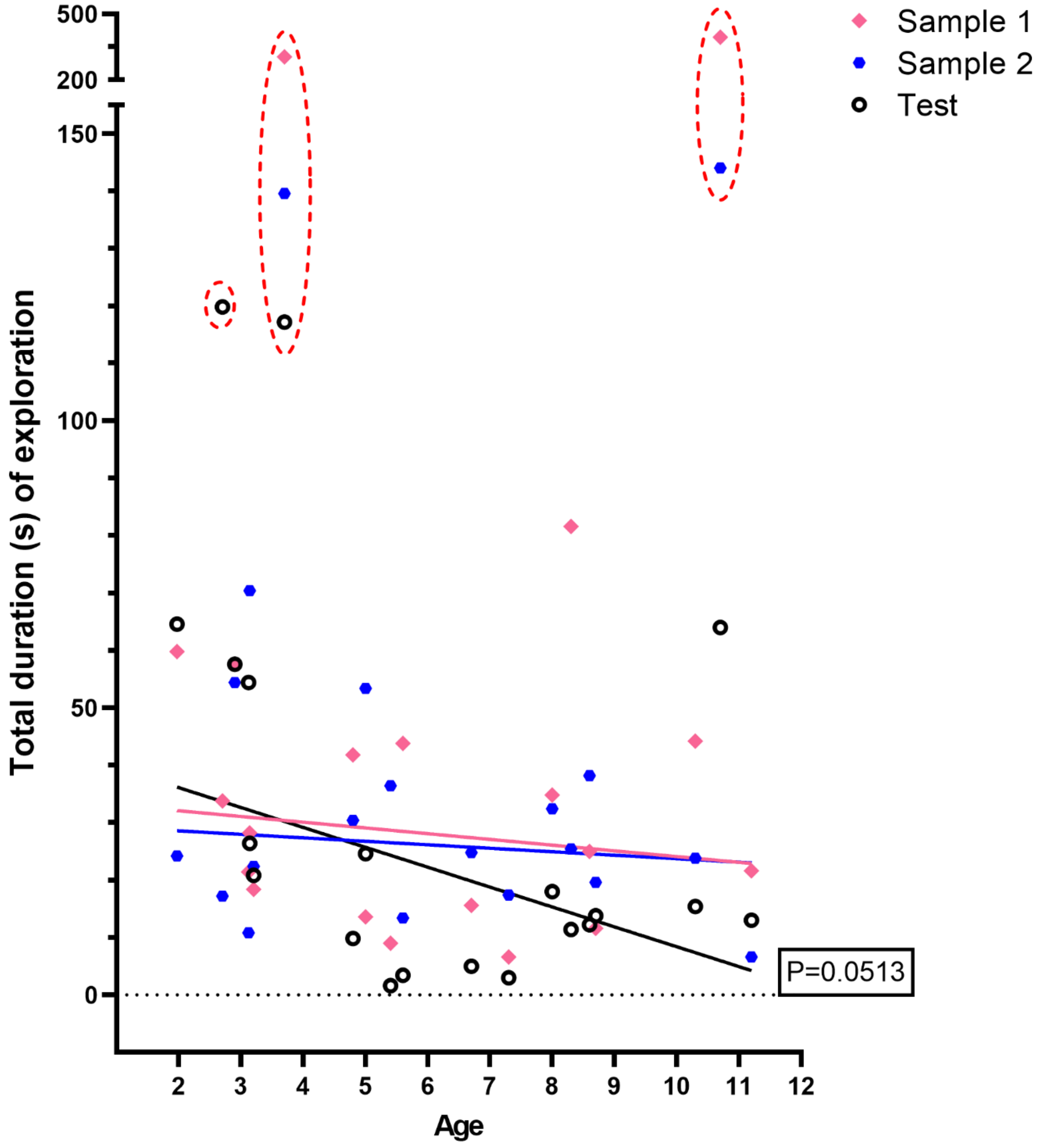

Supplementary *Figure S1. Exploratory behaviour in the www-task.* *Individual values for the total duration (in seconds) of exploration in sample 1 ◆, sample 2 ● and test ○ . Straight lines represent the robust linear regression fit. Outliers ⬭ .*

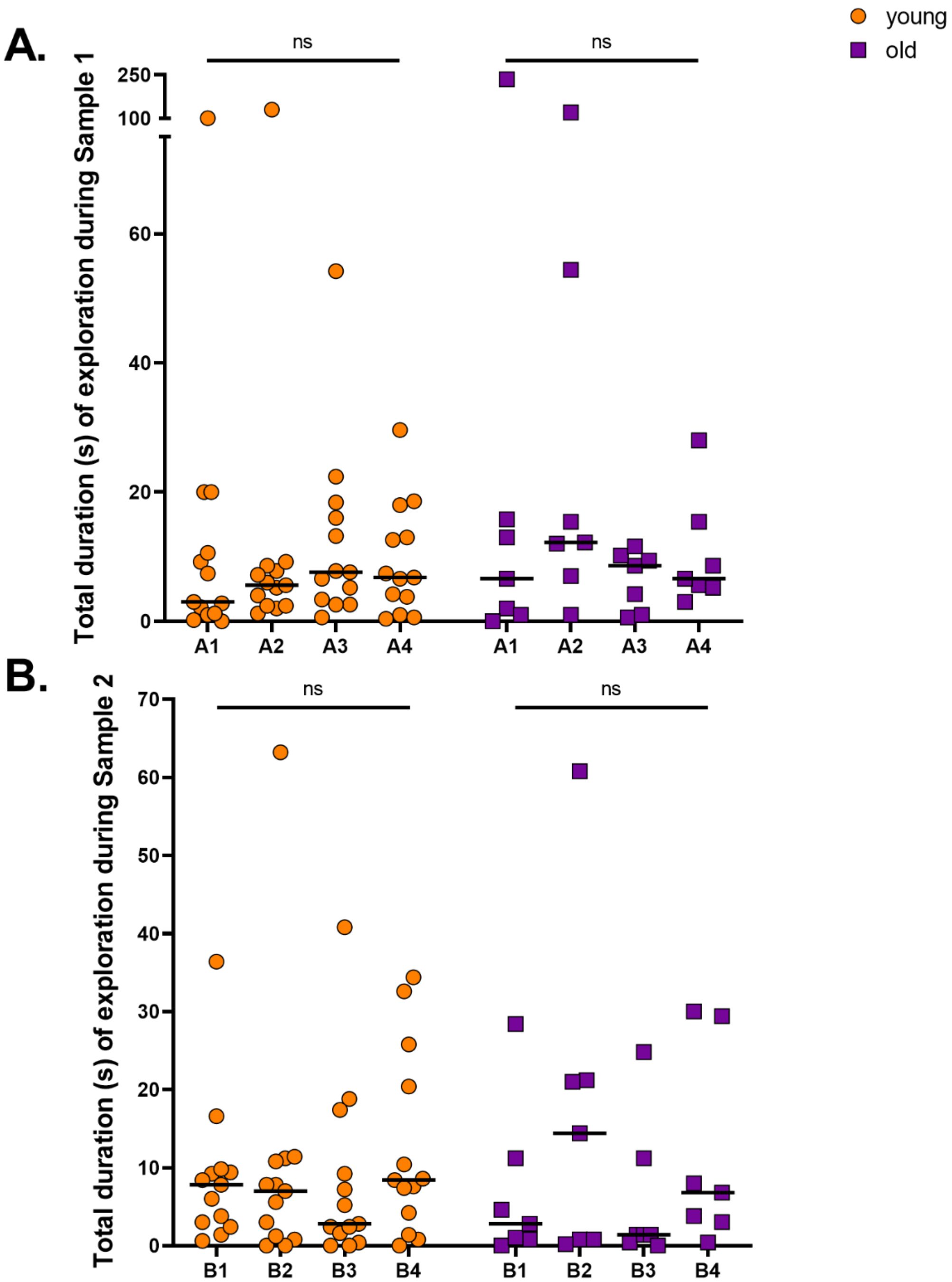

Supplementary *Figure S2*. ***Exploratory behaviour during the sample trials.*** *(A) Total time of exploration (duration in seconds) by the different groups (young; old) of each identical objects (A1-A4) during sample 1. (B) Total time of exploration (duration in seconds) by the different groups (same symbols as in A) of each identical objects (B1-B4) during sample 1. Individual values are shown. The horizontal line represents the group median; ns: not significant.*

Supplementary Table S1. *Duration of exploration (in seconds) during the different sample trials*

	Sample 1					Sample 2				
**Age**	**A1**	**A2**	**A3**	**A4**	**TT**	**B1**	**B2**	**B3**	**B4**	**TT**
1.97	9,2	7,8	13,2	29,6	**59,8**	9,4	7,8	2,8	4,2	**24,2**
2.7	2,8	5,2	7,8	18	**33,8**	6	0,8	0	10,4	**17,2**
2.9	20	8,6	16	13	**57,6**	9,2	5,6	5,2	34,4	**54,4**
3.12	2,2	9,2	3,4	6,6	**21,4**	0,6	7	2,4	0,8	**10,8**
3.14	3	6	6,6	12,6	**28,2**	7,8	11,2	18,8	32,6	**70,4**
3.2	7,4	2	5,2	3,8	**18,4**	3	11,4	0,4	7,6	**22,4**
3.7	101,4	130,8	54,2	18,6	**305**	9,8	63,2	40,8	25,8	**139,6**
4.8	20	2,4	18,4	1	**41,8**	8,4	0	1,6	20,4	**30,4**
5	0	5,6	0,6	7,4	**13,6**	16,6	10,8	17,4	8,6	**53,4**
5.4	1	1,2	2,6	4,2	**9**	36,4	0	0	0	**36,4**
5.6	10,6	4	22,4	6,8	**43,8**	1,4	1,2	2,4	8,4	**13,4**
6.7	0,2	7,2	7,6	0,6	**15,6**	2,4	7,8	7,2	7,4	**24,8**
7.3	1,2	2,4	2,6	0,4	**6,6**	3,8	3	9,2	1,4	**17,4**
8	15,8	15,4	0,6	3	**34,8**	0,8	0,8	1,4	29,4	**32,4**
8.3	13	54,4	8,6	5,6	**81,6**	0	21,2	0,4	3,8	**25,4**
8.6	2	12,2	4,2	6,6	**25**	4,6	14,4	11,2	8	**38,2**
8.7	1	1	1	8,6	**11,6**	11,2	0,2	1,4	6,8	**19,6**
10.3	6,6	12	10,2	15,4	**44,2**	1	21	1,4	0,4	**23,8**
10.7	235,2	120,4	11,6	28	**395,2**	28,4	60,8	24,8	30	**144**
11.2	0	7	9,4	5,2	**21,6**	2,8	0,8	0	3	**6,6**

Note: A1-A4: Set of identical objects presented during sample1. B1-B4: New set of identical objects presented during sample2. TT: Total time of exploration grouping the 4 objects together in each sample. Orange: young age group; purple: old age group.

Supplementary Table S2. Duration of exploration (in seconds) during the test trial.

	Test				
**Age**	**'Old' stationary**	**'Recent' stationary**	**'Old' displaced**	**'Recent' displaced**	**TT**
1.97	12,4	19	25,8	7,4	**64,6**
2.7	11,4	4,6	38,2	65,6	**119,8**
2.9	15,8	8	22,6	11,2	**57,6**
3.12	3	0	31,2	20,2	**54,4**
3.14	2,4	5	11	8	**26,4**
3.2	0,2	0,4	19,2	1	**20,8**
3.7	47	25,8	28	16,4	**117,2**
4.8	0,6	4,6	2,6	2	**9,8**
5	3,2	13,8	5,8	1,8	**24,6**
5.4	0	0	1,6	0	**1,6**
5.6	0	0	2,8	0,6	**3,4**
6.7	0	0	3,8	1,2	**5**
7.3	0	0	1,2	1,8	**3**
8	8	5,2	0,8	4	**18**
8.3	1,8	2	2,2	5,4	**11,4**
8.6	0	1,6	1,2	9,4	**12,2**
8.7	4,8	3	3,6	2,4	**13,8**
10.3	0,6	2	2	10,8	**15,4**
10.7	22,8	18,8	18,6	3,8	**64**
11.2	4	1,2	1,6	6,2	**13**

Note: 'Old' objects: objects A from sample 1. 'Recent' objects: objects B from sample 2. TT: Total time of exploration. Orange: young age group; purple: old age group.

Supplementary Table S3. Subjects kept for statistical analysis. Sex, age, behavioural experience and breeding situation.

Marmoset	Sex	Age (years)	Behavioral experience	Breeding situation
3696289	F	1,97	naïve	yes / n=6
3696273	M	2,7	touch screen / SD task	no / n=3
3696280	M	2,9	touch screen / SD task	no / n=3
3696270	M	3,12	touch screen / SD task	no / n=3
3696239	M	3,14	touch screen / SD task	no / n=2
3696278	F	3,2	naïve	yes / P / n=6
3696276	F	3,7	naïve	no / n=3
3696327	M	4,8	naïve	no / n=2
3696288	F	5	naïve	no / n=2
3592145	F	5,4	naïve	yes / P / n=6
3592143	M	5,6	Sadoun et al 2019	no / n=3
3592190	M	6,7	naïve	no / n=3
3592216	M	7,3	naïve	no / n=3
3287736	F	8	Sadoun et al 2019	no / n=2
3287706	M	8,3	Sadoun et al 2019 ($)	no / n=3
3287673	F	8,6	Sadoun et al 2019	no / n=3
3287755	M	8,7	Sadoun et al 2019	no / n=3
3287716	F	10,3	Sadoun et al 2019 ($)	no / n=2
1822	F	10,7	Sadoun et al 2019 ($)	no / n=3
1632	F	11,2	Sadoun et al 2019 ($)	no / n=2

Note: outliers are indicated by ^. ^^^ sample 1, sample 2 and test; ^ test. Blue and yellow indicate different families. P: Progenitor; n: total number of animals in the cage; SD: simple discrimination task. $ outliers in Sadoun et al., 2019.

Supplementary Table S4. Subjects excluded from the statistical analysis. Sex, age, behavioural experience and breeding situation.

Marmoset	Sex	Age (years)	Behavioral experience	Breeding situation
3696260	F	2,02	naïve	no / n=3
3696164	F	2,3	naïve	no / n=3
3696256	M	3,8	naïve	no / n=3
3592156	M	5,6	naïve	no / n=3
3592189	F	5,9	Sadoun et al 2019 ($)	no / n=3
3287697	M	7,1	Sadoun et al 2019 ($)	no / n=2
3287758	M	8,1	Sadoun et al 2019 ($)	no / n=3
1853	M	10,7	Sadoun et al 2019 ($)	no / n=2
1582	M	11,1	Sadoun et al 2019	no / n=2
1761	M	12	Sadoun et al 2019	no / n=2
1722	M	14,3	Sadoun et al 2019	no / n=3
1947	F	15	Sadoun et al 2019 ($)	no / n=2

Note: $ animals that showed cognitive deficits in DMTP in Sadoun et al., 2019. n: total number of animals in the cage.

Supplementary Table S5. Subjects' inter-observer correlations during the test trial.

Marmoset	Sex	Age (years)	Spearman correlation r (95% CI)	P (two-tailed) value
3696289	F	1,97	0.8631 (0.6729 to 0.9463)	P<0.0001
3696273	M	2,7	0.7721 (0.4900 to 0.9078)	P=0.0005
3696280	M	2,9	0.7893 (0.5228 to 0.9153)	P<0.0001
3696270	M	3,12	0.7658 (0.4782 to 0.9050)	P<0.0001
3696239	M	3,14	0.8178 (0.5789 to 0.9274)	P<0.0001
3696278	F	3,2	0.8196 (0.5828 to 0.9282)	P<0.0001
3696276	F	3,7	0.8321 (0.6079 to 0.9334)	P<0.0001
3696327	M	4,8	0.9368 (0.8404 to 0.9758)	P<0.0001
3696288	F	5	0.8044 (0.5523 to 0.9217)	P<0.0001
3592145	F	5,4	0.9986 (0.9963 to 0.9995)	P<0.0001
3592143	M	5,6	0.9160 (0.7909 to 0.9676)	P<0.0001
3592190	M	6,7	0.8587 (0.6635 to 0.9445)	P<0.0001
3592216	M	7,3	0.9908 (0.9756 to 0.9965)	P<0.0001
3287736	F	8	0.7498 (0.4487 to 0.8980)	P<0.0001
3287706	M	8,3	0.8939 (0.7404 to 0.9588)	P<0.0001
3287673	F	8,6	0.7702 (0.4864 to 0.9070)	P<0.0001
3287755	M	8,7	0.9589 (0.8914 to 0.9844)	P<0.0001
3287716	F	10,3	0.7952 (0.5341 to 0.9178)	P<0.0001
1822	F	10,7	0.9749 (0.9345 to 0.9905)	P<0.0001
1632	F	11,2	0.7988 (0.5412 to 0.9193)	P<0.0001
